# Optical monitoring of the plant growth status using polarimetry

**DOI:** 10.1038/s41598-022-26023-2

**Published:** 2022-12-17

**Authors:** Jongyoon Kim, Yu Kyeong Shin, Yunsu Nam, Jun Gu Lee, Ji-Hoon Lee

**Affiliations:** 1 Division of Electronics Engineering, Future Semiconductor Convergence Technology Research Center, Jeonbuk National University, Jeonju, 54896 Korea; 2 Department of Horticulture, College of Agriculture & Life Sciences, Jeonbuk National University, Jeonju, 54896 Korea; 3 Core Research Institute of Intelligent Robots, Jeonbuk National University, Jeonju, 54896 Korea; 4 Institute of Agricultural Science & Technology, Jeonbuk National University, Jeonju, 54896 Korea

**Keywords:** Plant sciences, Engineering, Optics and photonics

## Abstract

Polarimetry is a powerful characterization technique that uses a wealth of information from electromagnetic waves, including polarization. Using the rich information provided by polarimetry, it is being actively studied in biomedical fields such as cancer and tumor diagnosis. Despite its importance and potential in agriculture, polarimetry for living plants has not been well studied. A Stokes polarimetric imaging system was built to determine the correlation between the polarization states of the light passing through the leaf and the growth states of lettuce. The Stokes parameter *s*_3_ associated with circular polarization increased over time and was strongly correlated with the growth of lettuce seedlings. In the statistical analysis, the distribution of *s*_3_ followed the generalized extreme value (GEV) probability density function. Salt stress retarded plant growth, and the concentration of treated sodium chloride (NaCl) showed a negative correlation with the location parameter *μ* of GEV. The clear correlation reported here will open the possibility of polarization measurements on living plants, enabling real-time monitoring of plant health.

## Introduction

The polarization state of light refracted, reflected, or diffracted by a material depends on the properties of the material. Optical polarimetry is used to analyze the physical or chemical properties of materials by measuring and interpreting changes in the polarization state of light^1^. Ellipsometry is one of the well-known techniques used to characterize materials by measuring polarization states. The versatile power of polarization measurements is not limited to objects such as organic or inorganic compounds but can also be used in organisms (e.g., organs, tissues, and cells). Biomedical diagnostics using polarimetry is one of the most actively studied techniques in this field^2–5^. The huge benefit of polarimetric biomedical diagnostics is the noncontact and real-time measurement. For example, the Mueller polarimetric optical fiber is used for the diagnosis of cancers^4^.

For the same reason, diagnosis of living plants is a promising technique for agriculture. Meanwhile, there are few studies on polarization techniques for living plants^6–13^. Polarimetry uses not only the amplitude of an electromagnetic wave at a given wavelength of light but also the phase information^1^. Thus, it can provide more information about the plant than conventional hyperspectral analysis, which only uses the amplitude of the electromagnetic field^14–22^. Many parameters can be obtained from polarimetry, including the polarization azimuth angle and ellipticity. However, most previous research has been limited to using only depolarization information for monitoring the state of plants^6,8,12,13^. Thus, an exact correlation between the state of plants and the polarization state of light has not yet been revealed and remains an open issue.

Currently, some plants grow in controlled environments (e.g., greenhouses and plant factories) where light, temperature, and humidity are artificially regulated to allow for year-round harvesting. Meanwhile, plants can grow differently even in controlled environments, so monitoring techniques need to be developed. To determine the correlation between the polarization states and plant growth, a Stokes polarimetric imaging system was built. Using the Stokes imaging system, the polarization states of light passing through lettuce leaves were recorded and analyzed. Lettuce (*Lactuca sativa* L.) was chosen as the subject because it is one of the most popular leafy vegetables grown and consumed worldwide^23^. In this paper, a strong correlation between plant growth and polarization state was shown, proving the feasibility of polarization measurements for plant monitoring.

In addition, lettuce seedlings treated with different concentrations of sodium chloride (NaCl) were prepared, and the correlation between the salt level and the polarization states was studied. The growth of the plant affects the yield, and it can be retarded under stress conditions related to light quality, nutrition composition, water level, and salt concentration^24–27^. Among them, a moderate level of salt could enhance crop quality, while a high level of salt stress inhibits plant growth and results in mortality^28–32^. By using the proposed method, monitoring the growth of plants could be possible, including the level of salt stress.

## Results

### Stokes polarimetric imaging of lettuce

**Figure 1:**
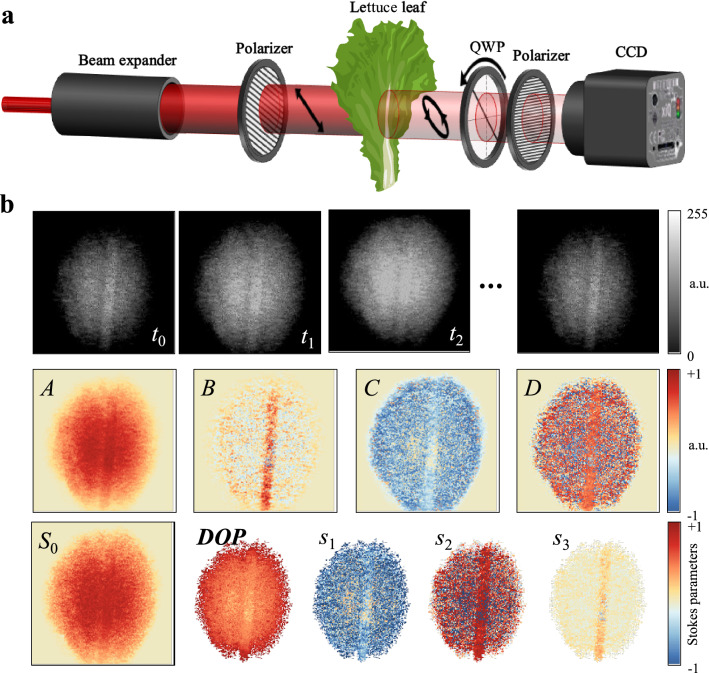
(**a**) Plant monitoring system using Stokes polarimetry. (**b**) Examples of processes to obtain the Stokes image. Each row corresponds to intensity, Fourier coefficients, and Stokes images in order of the first row to the third row.

Figure 1a shows a schematic of the plant monitoring system using Stokes polarimetry. The probe beam was incident on the CCD camera through a fixed polarizer with a transmission axis angle of 135°, a lettuce leaf, a rotating QWP ($\delta =\pi /2$), and a polarizer with $\alpha =0^\circ$. First, the intensity of the light passed through the lettuce leaf was recorded in a probe area of 25 mm diameter (first row of Fig. 1b). Each image is composed of the transmitted intensity in each pixel at the given time *t* while the QWP was rotating at a constant angular speed of 20 rad/s. The Fourier coefficients *A*, *B*, *C* and *D* were obtained by fitting the intensity at each pixel with Fourier expansion^33^ (second row in Fig. 1b). The Stokes parameters were calculated using the relationship between the Fourier coefficients and the Stokes parameters (third row in Fig. 1b). The degree of polarization (*DOP* = $\sqrt{{S}_{1}^{2}+{S}_{2}^{2}+{S}_{3}^{2}}/{S}_{0}$) was kept in a reasonable range (~ 70%) during the whole measurement process. Pixels with abnormal *DOP* (*DOP* ≤ 0% or *DOP* ≥ 100%) were excluded during the calculations. Most of the abnormal DOP data were shown due to the low light intensity of the beam edge. All sets of Stokes parameters were normalized to *S*_0_ = 1.

**Figure 2:**
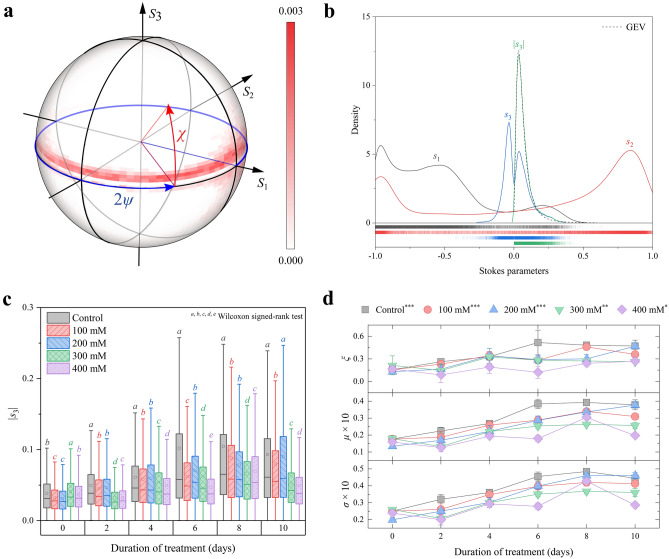
(**a**) Probability density plotted on the Poincaré sphere surface corresponding to polarization states of light after passing through the reference lettuce seedling leaves after 10 days of planting and (**b**) that of Stokes parameters obtained from the reference seedlings. (**c**) Box chart of |*s*_3_| and (**d**) means of GEV parameters as a function of treatment days for seedlings with different NaCl concentrations.

The Poincare sphere is a useful graphical tool to visualize the polarization states in the Cartesian coordinate system (*S*_1_*S*_2_*S*_3_-axes). Each polarization state (polarization ellipse) is uniquely given by the azimuth angles ψ and ellipticity χ and represented as a point on the Poincare sphere^1^. Figure 2a shows the probability density of polarization states, which was measured from the reference seedlings 10 days after planting. After passing through the lettuce leaf, most of the polarization states were placed near the equator (*S*_3_ = 0) and spread at azimuthal angles from 270° to 360°. The polarization states of the seedlings treated with NaCl also showed a similar distribution, making it difficult to distinguish the difference. Therefore, the Stokes parameter was statistically analyzed instead of the polarization state, as shown in Fig. 2b. The probability distribution of linear polarization components *s*_1_ and *s*_2_ was a pseudoexponential distribution and a superposition of two extreme distributions, respectively. Meanwhile, the circular polarization component *s*_3_ had two extreme distributions near 0, and it became single when applying the absolute to it (|*s*_3_|) and was easy to analyze.

The distribution of |*s*_3_| was changed by the NaCl concentration and duration of NaCl treatment, as shown in Fig. 2c. The difference between the distributions was tested by the Wilcoxon signed-rank test at a p-value < 0.001. Because the data did not meet a normal distribution, we used the Wilcoxon signed rank test, a nonparametric statistical hypothesis test. The median and median absolute deviation (MAD) of seedlings are summarized in Table 1. At the beginning of the measurement (day 0), the distribution of the reference and 400 mM seedlings was not significantly different from each of them, and the 100 mM and 200 mM seedlings were also statistically identical. After NaCl treatment, the |*s*_3_| distributions of the seedlings at the same duration of treatment became significantly different up to 4 groups for the durations of 2, 4, 8, and 10 days. All the distributions (five seedlings) were significantly different from each other at 6 days of treatment. The median and MAD tended to increase over time. Although the median of |*s*_3_| did not match the concentration of NaCl, it is meaningful that salt-stressed lettuce could be distinguished by measuring the Stokes parameters. If there are seedlings with a significantly lower median of |*s*_3_|, they can be considered salt-stressed. For example, the median of control seedlings was always higher than that of other seedlings, but the median of seedlings treated with 400 mM NaCl was smaller than that of other seedlings.

**Table 1:** Summary of statistical analysis of *s*_3_ obtained by measuring seedlings.

Treatments	Days	Median	MAD*	*ξ*		*μ*		*σ*	
				M	SD	M	SD	M	SD
Control	0	0.0312	0.0156	0.1558	0.0509	0.0176	0.0005	0.0250	0.0009
	2	0.0385	0.0186	0.2628	0.0362	0.0224	0.0020	0.0320	0.0024
	4	0.0458	0.0224	0.3294	0.0392	0.0268	0.0011	0.0360	0.0010
	6	0.0579	0.0332	0.5178	0.1461	0.0385	0.0025	0.0455	0.0024
	8	0.0653	0.0355	0.4830	0.0274	0.0392	0.0009	0.0484	0.0013
	10	0.0611	0.0346	0.4712	0.0694	0.0379	0.0026	0.0445	0.0018
100 mM	0	0.0277	0.0125	0.1558	0.0509	0.0176	0.0005	0.0250	0.0009
	2	0.0342	0.0162	0.2301	0.0078	0.0187	0.0031	0.0261	0.0029
	4	0.0432	0.0211	0.3443	0.0498	0.0259	0.0019	0.0348	0.0016
	6	0.0487	0.0240	0.2828	0.0128	0.0288	0.0018	0.0397	0.0027
	8	0.0585	0.0316	0.4601	0.0224	0.0340	0.0017	0.0420	0.0018
	10	0.0554	0.0280	0.3592	0.0350	0.0309	0.0012	0.0413	0.0009
200 mM	0	0.0268	0.0112	0.1298	0.0176	0.0134	0.0008	0.0199	0.0013
	2	0.0349	0.0174	0.1628	0.0537	0.0169	0.0012	0.0250	0.0010
	4	0.0430	0.0225	0.3406	0.0902	0.0221	0.0032	0.0302	0.0026
	6	0.0540	0.0267	0.2911	0.0487	0.0287	0.0023	0.0398	0.0019
	8	0.0576	0.0285	0.3005	0.0505	0.0336	0.0009	0.0460	0.0013
	10	0.0596	0.0335	0.4686	0.0307	0.0377	0.0015	0.0461	0.0016
300 mM	0	0.0331	0.0151	0.2143	0.1127	0.0179	0.0015	0.0260	0.0009
	2	0.0262	0.0109	0.1459	0.0779	0.0131	0.0014	0.0207	0.0014
	4	0.0404	0.0198	0.3258	0.0199	0.0220	0.0012	0.0300	0.0017
	6	0.0457	0.0222	0.2836	0.0279	0.0253	0.0012	0.0351	0.0014
	8	0.0510	0.0243	0.2723	0.0151	0.0264	0.0008	0.0368	0.0012
	10	0.0424	0.0193	0.2633	0.0108	0.0257	0.0016	0.0361	0.0021
400 mM	0	0.0315	0.0140	0.1646	0.0255	0.0158	0.0007	0.0238	0.0008
	2	0.0277	0.0118	0.0870	0.0892	0.0126	0.0022	0.0200	0.0028
	4	0.0373	0.0172	0.1915	0.0783	0.0193	0.0006	0.0293	0.0010
	6	0.0376	0.0164	0.1215	0.0689	0.0179	0.0017	0.0279	0.0017
	8	0.0535	0.0268	0.2414	0.0306	0.0304	0.0010	0.0428	0.0014
	10	0.0383	0.0175	0.2686	0.0133	0.0197	0.0007	0.0286	0.0010

*Median absolute deviation.

To find a clearer correlation between the plant state and the polarization state, we tried to fit the data with the generalized extreme value (GEV) distribution. The GEV distribution is widely used to model economic or natural events such as financial risk and rainfall^34^. The probability density function of the GEV is given by,

1 $$f\left(s;\xi \right)=\left\{ \begin{array}{ll}\mathit{exp}\left(-s\right)\mathit{exp}\left[\mathit{exp}\left(-s\right)\right],& \xi =0\\ {\left(1+\xi s\right)}^{-(1+1/\xi )}\mathit{exp}\left[-{\left(1+\xi s\right)}^{-1/\xi }\right],& \xi \ne 0 \;\; and \;\; \xi s>-1\\ 0,& otherwise\end{array}\right.$$

where *s* = (*x*-*μ*)/*σ*, *μ* is the location parameter, *σ* is the scale parameter, and *ξ* is the shape parameter^34^. The probability density distribution of |*s*_3_| was well matched with the GEV, as shown in Fig. 2b. The average GEV parameters of the five replicants are shown in Fig. 2d, where the error bar is the standard deviation. There seems to be a positive linear correlation between GEV parameters and the duration of treatment, while the correlation was weaker at higher NaCl concentrations. The asterisk symbols ***, **, and * in the legend of Fig. 2d represent the significance of *ξ* at p-value < 0.05, 0.01, 0.001 obtained by analysis of variance (ANOVA). The shape parameter ξ of the reference, 100 mM, and 200 mM seedlings was significant at p-value < 0.001 (***) depending on the duration of treatment. The significance levels of seedlings treated with 300 mM and 400 mM NaCl were 0.01 (**) and 0.05 (*), respectively. Therefore, a high concentration of NaCl interrupted the change in the circular polarization component (*s*_3_) over time. A similar trend was also shown in the summary statistics of seedlings treated with 400 mM NaCl (Fig. 2c).

**Figure 3:**
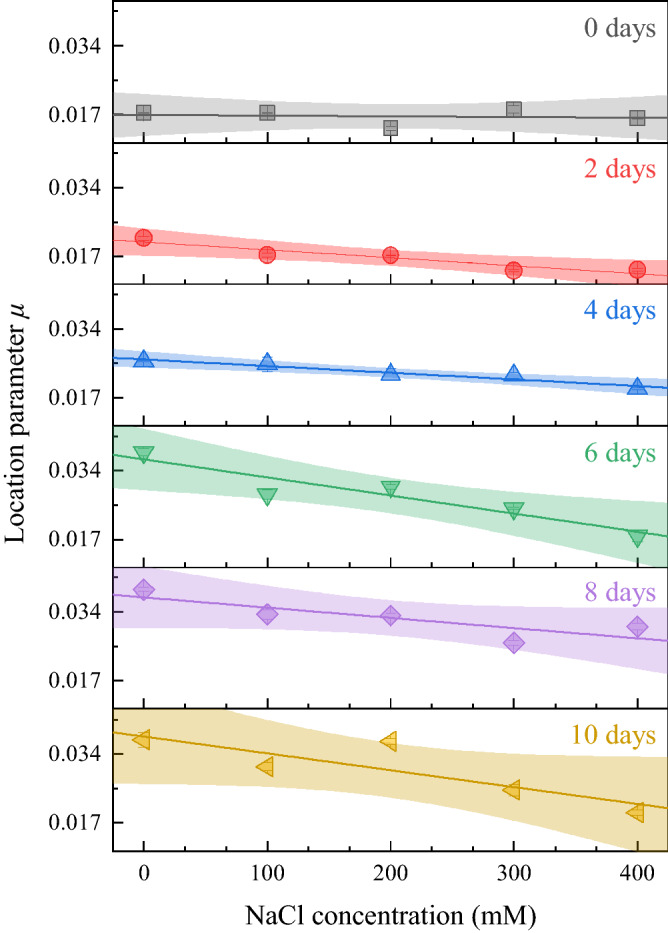
Median (symbols) and linear regression (lines) with 95% confidence range (shades) of location parameter *μ* for five replicants with different durations of treatments as a function of the concentration of NaCl.

After obtaining the GEV parameters, a statistical correlation analysis was performed. The Spearman correlation between the concentration of NaCl and the location parameters showed the greatest correlation (Fig. S3). Figure 3 shows the results of linear regression on the median of |*s*_3_| for each duration of treatment as a function of the concentration of NaCl, where the error bar is the MAD. Before the start of the treatment (0 days), the location parameters were not correlated with the concentration of NaCl. Very after salt stress (2 days), a negative linear correlation was clearly shown, and the negative slope became greater with durations until 10 days. Therefore, the salt stress level of lettuce grown during the same period could be determined by measuring the Stokes parameter. This result could be applied to lettuces grown for different durations of time because the growth stages of the seedlings could be determined by summary statistics (Fig. 2c).

**Figure 4:**
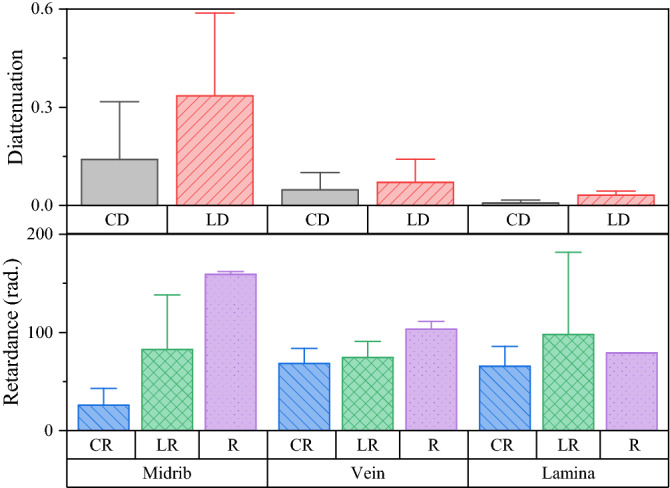
Polarization components obtained from a Mueller matrix of a lettuce leaf, where CD and LD are the circular and linear diattenuations, CR and LR are circular and linear retardances and R is the total retardance.

The polarization of light can be changed by optical characteristics such as birefringence, diattenuation, and depolarization. Figure 4 shows the polarization response of lettuce obtained by Mueller matrix decomposition. The values are the average of three measurements, and the uncertainty is the standard deviation. The diattenuation was large at the midrib, while those in the vein and the lamia were small. Meanwhile, the retardation, especially linear retardation, was high in all parts of the leaf. Most of the light passed through the lamia during the experiments. In the lamia region, the retardation is much larger than the diattenuation. Therefore, the change in circular polarization components after passing through the lettuce seedlings is probably related to the optical retardation of the lettuce. Optical retardation in plants has been reported in many works in the literature^9–11^. For example, linear retardation can be shown by the aligned cellulose in the plant, and glucose or layer structures could produce circular retardation^35–40^. As the plant grows, the physical properties (growth parameters) of the leaves increase, e.g., length, weight, and density. Linear retardance is simply given by the product of birefringence and thickness. The birefringence is known to depend on the density so that it would be increased while the plant is growing. Therefore, the linear retardation will naturally increase as well-grown plants have thicker leaves.

Salt stress is known to hinder the growth of plants, including lettuce. The growth of lettuce seedlings retards with a high concentration of NaCl (Figs. S1, S2). The circular polarization components tended to increase with the growth of the seedling over time (Fig. 2c). The degeneration of growth results in the inhibition of the formation of lettuce cells, including cellulose and other retardation-related parts. Thus, the level of salt stress was shown as the change in the circular polarization component (*s*_3_) of the light passed through the lettuce.

## Discussion

The optical response of lettuce was studied, and circular polarization (*s*_3_) was found to be sensitive to the growth of the seedlings. The polarization of light passed through the leaves of the lettuce seedlings was measured by the Stokes imaging system. While the seedlings were growing, the median and the variance of the circular polarization components of the Stokes parameters (*s*_3_) were increased. The growth of the seedlings was significantly retarded by the high concentration of NaCl. As a result, the circular polarization did not change significantly as the salt stress level increased. The probability density of *s*_3_ was well matched with the GEV probability density function, and the GEV parameters then increased over the growth time. Among the GEV parameters, the location parameter (*μ*) showed the greatest Spearman correlation with the concentration of NaCl. The change in *s*_3_ seems to be related to the retardation components of the plants, such as linear retardation from cellulose or circular retardation of glucose.

These results will be helpful for developing a remote sensing system for monitoring the growth (or health) of plants. Compared with the hyperspectral technique, the polarimetry data are much more compact to analyze because a single wavelength is used as in this paper. In addition, Stokes polarimetry requires only the polarization state analyzer, so it has a straightforward configuration compared to Mueller polarimetry. The great statistical significance between *s*_3_ and the growth time or the concentration of NaCl is shown. Therefore, the classification of the level of growth and salt stress will be possible with the assistance of machine learning.

## Materials and methods

### Stokes parameters and Mueller matrix

The configuration and process of Stokes polarimeters using a rotating retarder with a fixed polarizer have been well established by various studies^41–43^. In the configuration shown in Fig. 1a, the intensity of arbitrarily polarized light incident on a detector (or pixel) can be rewritten as,

2 $$I=\frac{1}{2}[A-B\;\mathit{sin}\;2\left(\omega t+{\varphi }_{0}\right)+C\;\mathit{cos}\;4\left(\omega t+{\varphi }_{0}\right)+D\;\mathit{sin}\;4(\omega t+{\varphi }_{0}) ]$$

where $\omega$ is the rotational velocity and ${\varphi }_{0}$ is the initial phase. Using Fourier expansion of the light intensity, the Stokes parameters are given by^41^,

3 $${S}_{0}=A-C, {S}_{1}=2C, {S}_{2}=2D, {S}_{3}=B$$

The recorded light intensity of each pixel was fitted by Fourier expansion, and then Fourier coefficients and Stokes parameters were obtained.

The Mueller matrix of the lettuce was measured by a commercial Mueller matrix polarimeter AxoScan (AxoMetrics).

### Image processing and statistical analysis

Stokes images were image processed and fitted by using MATLAB (MathWorks). Then, the processed data were statistically analyzed by using RStudio Desktop (RStudio).

### Preparation of the lettuce samples

Lettuce (*Lactuca sativa* L.) cultivar ‘Cheong Chi Ma’ (Asia Seed) seeds were sown in 50-cell plug trays (54.4 cm × 28.2 cm × 5.4 cm) filled with soil, and the trays were kept inside a lightbox (65 cm × 35 cm × 50 cm). The seedlings were grown under a fluorescent lamp (TLD 32 W/865RS, Philips) where the photosynthetic photon flux density (PPFD) was 210 ± 10 μmol/m^2^ s^−1^ and the photoperiod was 16 h light and 8 h dark at 24 °C and 18 °C, respectively. The relative humidity was kept at 50% during the experiments. The seedlings were subirrigated with tap water for 20 min each morning for 12 days and then treated with NaCl solution for 10 days. The treatment solutions were prepared by dissolving NaCl at concentrations of 100, 200, 300, and 400 mM in tap water. Seedlings treated with only water (0 mM) were used as controls.

### Ethical approval

The authors confirm that no plant or seed specimens were collected in this study. The seeds were purchased from Asia Seed in South Korea, and all plant materials were grown in the author's laboratory. This study complies with relevant institutional, national, and international guidelines and laws, and no genotyping data were analyzed or generated during the study.

## Supplementary Information

Supplementary Figures.

## Data Availability

Data underlying the results presented in this paper are not publicly available at this time but may be obtained from the authors upon reasonable request. To request the data, please contact the corresponding author.
