# Supplementary material for: Optical monitoring of the plant growth status using polarimetry
Source: Sci Rep. 2022 Dec 17;12:21841. doi: 10.1038/s41598-022-26023-2 (PMC9759557; doi:10.1038/s41598-022-26023-2)
Supplement: Supplementary file 1 — Supplementary Figures. [file 41598_2022_26023_MOESM1_ESM.docx]

**Supplementary Information for Optical monitoring of the plant growth status using the polarimetry**

Jongyoon Kim^1^, Yu Kyeong Shin^2^, Yun Su Nam^1^, Jun Gu Lee^2,3^ and Ji-Hoon Lee^1*^

*^1^Future Semiconductor Convergence Technology Research Center, Division of Electronics Engineering, Jeonbuk National University, Jeonju 54896, Korea*

^2^Department of Horticulture, College of Agriculture & Life Sciences, Jeonbuk National University, Jeonju 54896, Korea

^3^Institute of Agricultural Science & Technology, Jeonbuk National University, Jeonju 54896, Korea

* Corresponding author: jihoonlee@jbnu.ac.kr


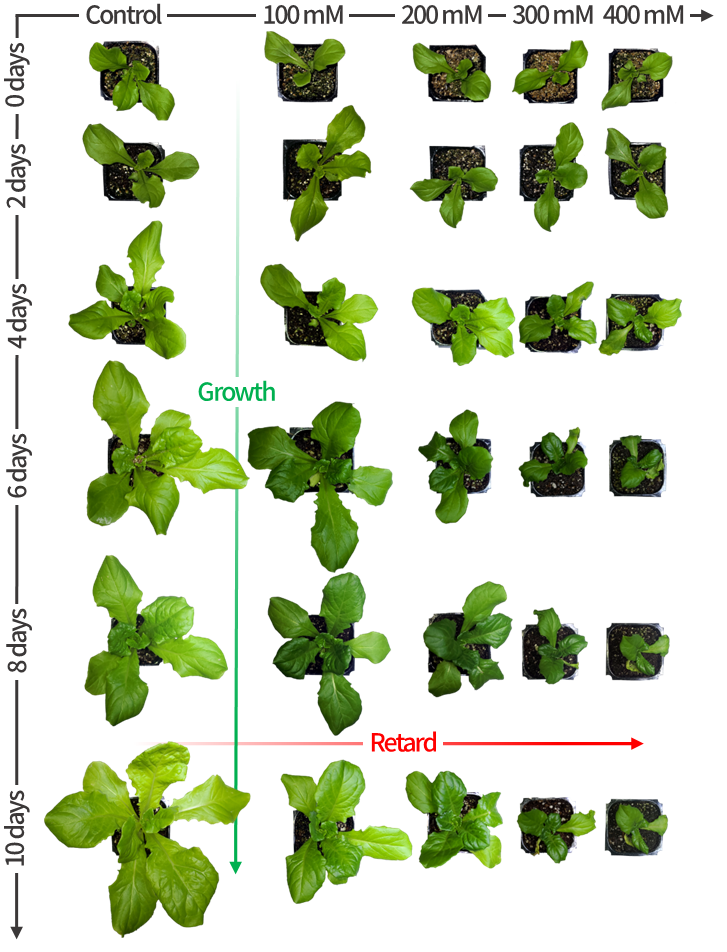


Fig. S1. Visual appearances of the seedlings as they grew up under different concentrations of NaCl.


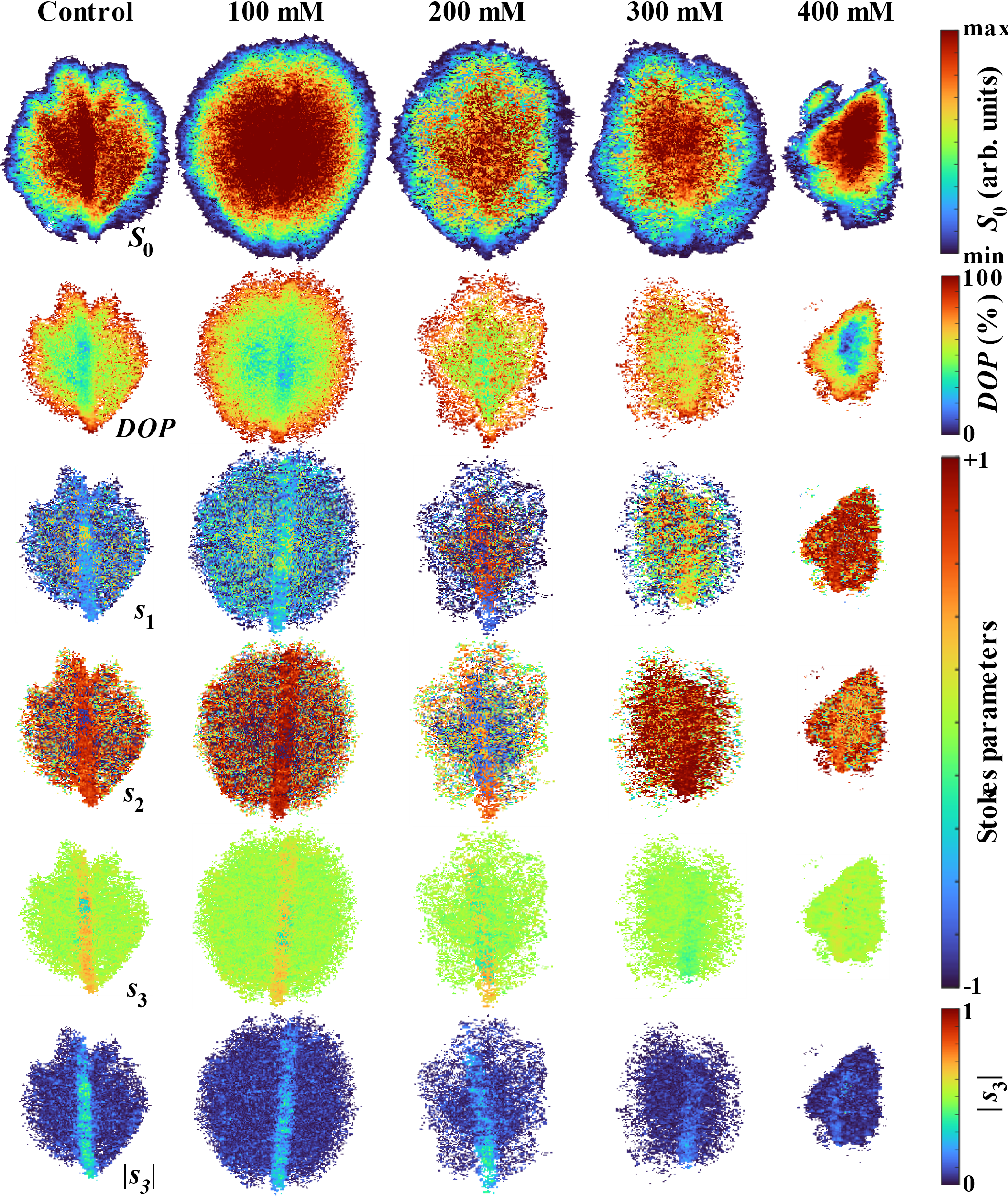


Fig. S2. Stokes polarimetric images of the lettuce seedlings with different concentration of NaCl after 10 days of treatment.


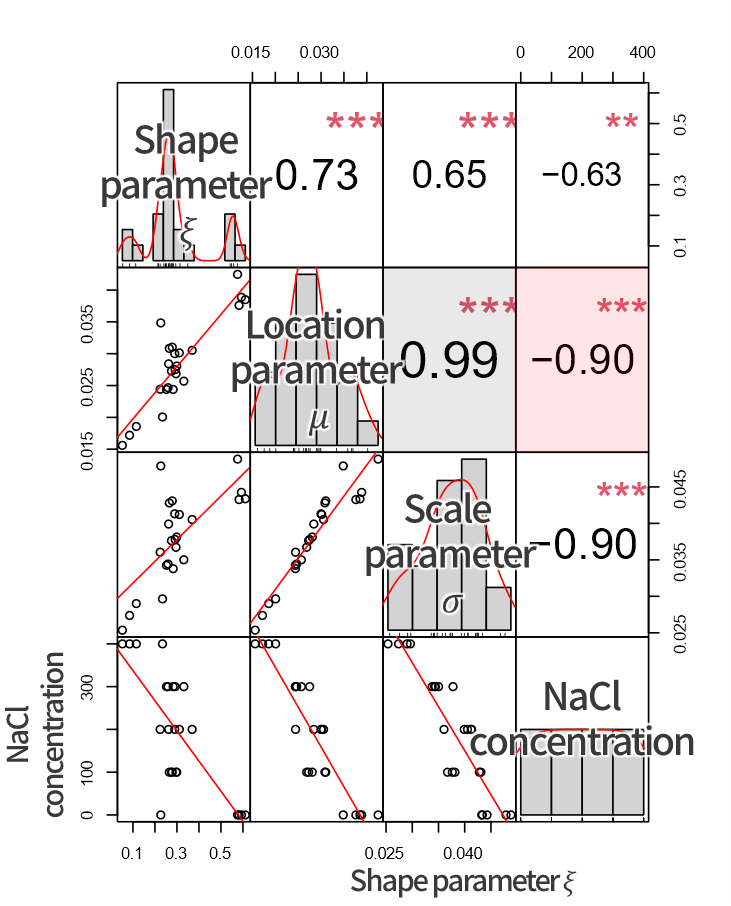


Fig. S3. Correlation matrix charts of the GEV parameters (𝜉, 𝜇, 𝜎) and the NaCl concentration at 6 days of the treatment. The numbers in the charts are Spearman’s rank correlation coefficient and asterisks (*, **, ***) are the significance at p-value < 0.05, 0.01, 0.001.
